# Study of the serotonin transporter (*SLC6A4*) and *BDNF *genes in French patients with non syndromic mental deficiency

**DOI:** 10.1186/1471-2350-11-30

**Published:** 2010-02-22

**Authors:** Refaat Tabagh, Christian R Andres, Sylviane Védrine, Catherine Cherpi-Antar, Rose-Anne Thepault, Laurence Mignon, Diane Dufour-Rainfray, Claude Moraine, Patrick Vourc'h

**Affiliations:** 1 UMR Inserm U930, Université François Rabelais de Tours, Tours, France; 2 Laboratoire de Biochimie et Biologie moléculaire, CHRU de Tours, Tours, France; 3 Neuroscience Education Institute, Carlsbad, CA, USA

## Abstract

**Background:**

Mental deficiency has been linked to abnormalities in cortical neuronal network connectivity and plasticity. These mechanisms are in part under the control of two interacting signalling pathways, the serotonergic and the brain-derived neurotrophic (BDNF) pathways. The aim of the current paper is to determine whether particular alleles or genotypes of two crucial genes of these systems, the serotonin transporter gene (*SLC6A4*) and the brain-derived neurotrophic factor gene (*BDNF*), are associated with mental deficiency (MD).

**Methods:**

We analyzed four functional polymorphisms (rs25531, 5-HTTLPR, VNTR, rs3813034) of the *SLC6A4 *gene and one functional polymorphism (Val66 Met) of the *BDNF *gene in 98 patients with non-syndromic mental deficiency (NS-MD) and in an ethnically matched control population of 251 individuals.

**Results:**

We found no significant differences in allele and genotype frequencies in the five polymorphisms studied in the *SLC6A4 *and *BDNF *genes of NS-MD patients versus control patients. While the comparison of the patterns of linkage disequilibrium (D') in the control and NS-MD populations revealed a degree of variability it did not, however, reach significance. No significant differences in frequencies of haplotypes and genotypes for VNTR/rs3813034 and rs25531/5-HTTLPR were observed.

**Conclusion:**

Altogether, results from the present study do not support a role for any of the five functional polymorphisms of *SLC6A4 *and *BDNF *genes in the aetiology of NS-RM. Moreover, they suggest no epistatic interaction in NS-MD between polymorphisms in *BDNF *and *SLC6A4*. However, we suggest that further studies on these two pathways in NS-MD remain necessary.

## Background

Mental deficiency (MD) is defined as an intelligence quotient (IQ) below 70 with impairments in social skills, self-care, and work. The prevalence of non-syndromic-MD (NS-MD) in developed countries is higher than 0.5% [1,2]. A large proportion of NS-MD cases results from abnormalities in neuronal network connectivity and plasticity, and these require particular neurotrophic factors and neurotransmitters for proper establishment and functioning [3].

**Figure 1:**
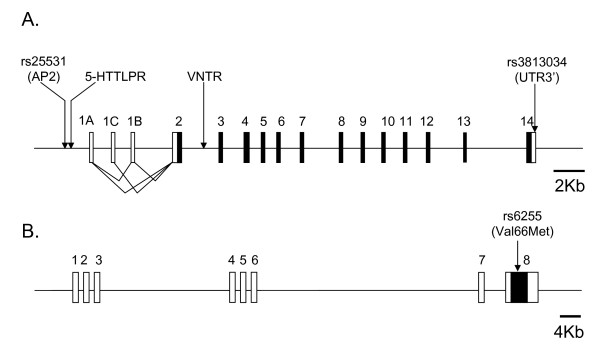
**Schematic structure of the human SLC6A4 (A) and BDNF (B) genes**. Non-coding and coding exons are indicated by white and black boxes, respectively. Locations of the genotyped polymorphisms and alternative splicing are indicated.

Serotonin (5-hydroxytryptamine, 5-HT) is a major neurotransmitter involved in several complex behaviours, including cognition and emotion, which have been shown to be altered in NS-MD [4]. Pharmacological and genetic studies also support a role for serotonin in corticogenesis, and brain development in general, even prior to the formation of synapses [5-8]. One key regulator of serotonin levels in the cortex and hippocampus is the serotonin transporter (5-HTT), which is involved in the reuptake of extracellular serotonin [9,10]. 5-HTT is a Na^+^/Cl^- ^dependant membrane transporter encoded by the *SLC6A4 *gene (solute carrier family 6 member 4; 17q11.2) (Figure 1) [11]. A polymorphism (5HTTLPR) in the promoter region of SLC6A4 determines the neuroanatomical size and functional coupling of the amygdala-frontal cortical circuit in humans [12,13]. This circuit has been implicated in several psychiatric disorders including Fragile X and Williams syndrome, two pathologies characterized by mental deficiency [14,15].

The expression level of 5-HTT is affected by several polymorphisms in the *SLC6A4 *gene (Figure 1). In the promoter region, both the rs25531 polymorphism (SNP A/G), which is part of a putative binding domain for the AP2 transcription factor, and the 5HTTLPR polymorphism (deletion/insertion) influence the expression levels of 5HTT [16,17]. A neurophysiological imaging study in humans described an association between 5-HTTLPR and prefrontal cortex-limbic excitability, supporting a relation between 5-HTT and cognitive processing [18,19]. 5HTT expression can also be affected by a variable number of tandem repeats (VNTR) in intron 2 and a SNP (rs3813034) in the 3'UTR of the *SLC6A4 *gene [20,21].

The function of 5HTT is modulated by brain-derived neurotrophic factor (BDNF) as confirmed by experiments on BDNF-deficient mice [22,23]. BDNF, a member of the growth factor family of neurotrophins, contributes to the activity-dependent synaptic development and survival of serotonergic neurons [9,24]. In general, BDNF is known to have crucial roles during brain development as well as in adults by regulating synaptic transmission and plasticity. Indeed, modulation of BDNF expression in mice affects synaptic vesicle functioning and plasticity, leading to defects in spatial learning [25]. The secreted protein, BDNF, is encoded by the *BDNF *gene located at 11p13 (Figure 1) [26]. The SNP rs6255 (Val66 Met) in this gene affects the activity-dependent secretion of BDNF as well as short-term episodic memory in humans [27,28].

When combined with the observation of abnormal serotonin and BDNF blood concentrations in several subjects with NS-MD, the evidence presented thus far suggets that the serotonergic and BDNF signalling systems may be implicated in the aetiology of NS-MD [29-31]. We therefore tested this hypothesis by analysing five functional polymorphisms in the *SLC6A4 *and *BDNF *genes in patients with NS-MD.

## Methods

### Subjects

The patients with NS-MD (n = 98; 77 males and 21 females) were recruited in the genetics unit of the University hospital in Tours (France). All patients showed moderate to severe NS-MD and were submitted to a careful clinical examination, searching for specific signs of the NS-MD syndrome. The patients had no familial history of MD and were therefore considered sporadic. All were negative for fragile X mutation (FRAXA). The control group (n = 251) consisted of anonymous and unrelated French Caucasian individuals without MD. DNA was extracted from peripheral blood sample using a standard protocol. All patients or parents gave their informed consent.

### Genotyping

The rs25531 and 5-HTTLPR in *SLC6A4 *were analyzed by RFLP as described in Wendland et al. [32]. The PCR amplification step allowed to differentiate between the short (S, 484 bp) versus the long allele (L, 528 bp) of 5-HTTLPR. The restriction step using HpaII enzyme (Biolabs) permitted to identify the alleles (A/G) of rs25531, located 19 bp upstream of 5-HTTLPR. Thus, 10 patterns of migration corresponding to 10 genotypes (rs25531/5-HTTLPR) could be identified after electrophoresis on a 2% agarose gel (all primers and conditions are available upon request).

The VNTR (17 bp variable number of tandem repeats) in SLC6A4 was studied by PCR followed by electrophoresis on 3% agarose gel stained with ethidium bromide. PCR conditions for the amplification of the 9 (345 bp), 10 (360 bp) and 12 (390 bp) repeat alleles are described in Cook et al. [33].

The rs3813034 (G/T) in the 3'UTR region of *SLC6A4 *gene was genotyped by RFLP. PCR was performed on 100 ng of genomic DNA with 125 μM of each dNTP, 200 nM of primers, 1.5 mM MgCl_2 _and 0.02 U/μL of Taq DNA polymerase (Invitrogen), before incubation with MseI (Fermentas) and electrophoresis on a 2% agarose gel.

The rs6255 (G/A) in *BDNF *was studied by denaturing high performance liquid chromatography (dHPLC, WAVE System 3500 HT, Transgenomic). PCR was performed on 100 ng of genomic DNA with 125 μM of each dNTP, 200 nM of primers, 1.5 mM MgCl_2 _and 0.02 U/μL of Taq DNA polymerase (Invitrogen) (Tm: 59°C). PCR products were denaturated (96°C 5 min) and slowly renaturated (96°C to 50°C; 1°C/minute) before analysis by dHPLC at 60.2°C. For individuals showing only homoduplex formations (G/G or A/A homozygotes), 10 μL of PCR were mixed with 10 μL of a PCR product (standard) previously analyzed by DNA sequencing (G/G homozygote). The mixture was denaturated, renaturated, and analysed by dHPLC.

### Statistical analysis

Allele and genotype frequencies in patients and controls were compared with a χ^2 ^test. Odds ratios were estimated for alleles and genotypes http://www.hutchon.net/ConfidOR.htm[34]. Linkage disequilibrium (parameter D') between markers was calculated using the software Haploview v.3.32, with the exception of rs25531 and 5HTTLPR polymorphisms for which chromosome phases could be obtained directly. LD patterns were analysed by Spearman rank correlation (Excel) between LD measures.

## Results

### Analysis of SLC6A4 polymorphisms in NS-MD

**Figure 2:**
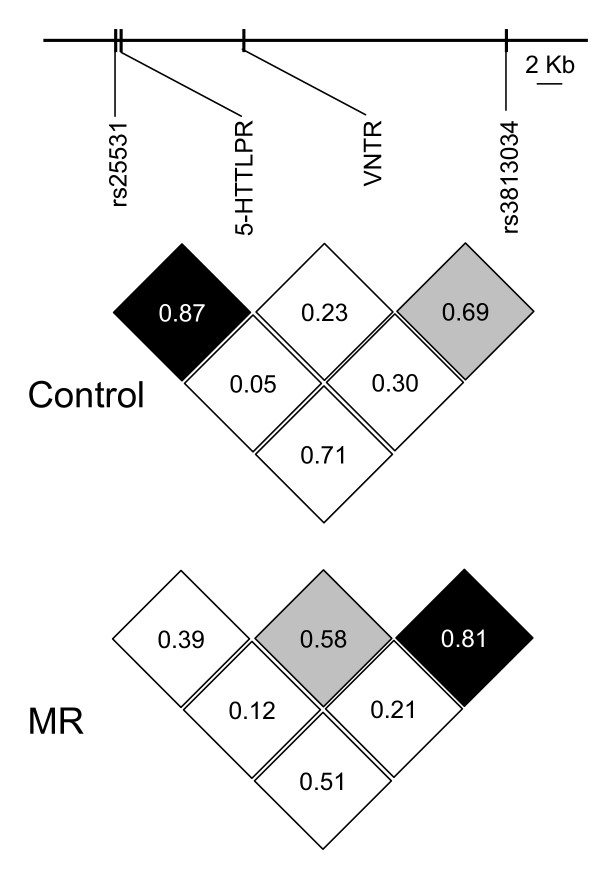
**Linkage disequilibrium (LD) map of four polymorphic markers in SLC6A4 gene in control and NS-MD patients**. In each square, the normalized linkage disequilibrium measures (D') corresponding to each pair of markers are indicated. Squares are in black if the D' values are ≥ 80% (strong LD) and in grey if the D' values are between 50-80%.

Hardy Weinberg distribution was respected for the four polymorphisms in *SLC6A4 *in the two populations (not shown). We did not observe a significant association between NS-MD and the rs25531 (χ^2 ^= 0.98; p = 0.32), 5-HTTLPR (χ^2 ^= 0.07; p = 0.79) VNTR (χ^2 ^= 0.51; p = 0.47), and rs3813034 (χ^2 ^= 0.69; p = 0.40) alleles (Figure 1; table 1). Similarly, no significant association was detected between NS-MD and the genotypes of the four polymorphisms (data not shown). The genotyping method used for rs25531 (AP2) and 5-HTTLPR permitted a direct identification of the phased genotypes. Using these phased genotypes, no significant association between NS-MD and a particular haplotype (χ^2 ^= 0.99; p = 0.61) or genotype (χ^2 ^= 1.13; p = 0.77) was detected. As Hu et al. (2006) reported that the GL genotype can drive 5HTT expression nearly equivalently to any S genotype (AS or GS), we consequently grouped the GL, AS, and GS haplotypes for analysis. However we still did not observe an association between NS-MD and the haplotype (χ^2 ^= 0,12; p = 0.73) or genotype (χ^2 ^= 1.28; p = 0.73) (Table 2). We used pairwise LD measures (D') between adjacent markers to analyse the patterns of LD in the control and NS-MD populations (Figure 2). Comparison of these unrelated populations revealed a degree of variability which, however, did not reach significance (Spearman rank correlation, r_s _= 0.50; p = 0.66). We next analyzed the VNTR/rs3813034 haplotypes in the two populations, and again found no significant difference for haplotype (χ^2 ^= 3.80; p = 0.28) and genotype (χ^2 ^= 3.50; p = 0.75) distributions between controls and NS-MD patients (Table 3).

**Table 1:** Allelic distributions of polymorphisms in SLC6A4 and BDNF genes in the control and NS-MD populations.

Markers	Alleles	Controls	MR patients	MR vs Controls	
				
				*χ*^2^	p
SLC6A4 gene					
rs25531	A	308 (93.9)	188 (95.9)	0.98	0.32
	G	20 (6.1)	8 (4.1)		
5-HTTLPR	L	198 (60.4)	116 (59.2)	0.07	0.79
	S	130 (39.6)	80 (40.8)		
VNTR	9	4 (1.2)	2 (1.0)	0.60	0.74
	10	113 (34.5)	74 (37.8)		
	12	211 (64.3)	120 (61.2)		
rs3813034	T	187 (57.0)	119 (60.7)	0.69	0.40
	G	141 (43.0)	77 (39.3)		
BDNF gene					
rs6255	G	378 (75.2)	129 (77.7)	0.40	0.53
	A	124 (24.8)	37 (22.3)		

**Table 2:** Haplotypes and phased genotypes distributions for the rs25531 and 5-HTTLPR polymorphisms in the SLC6A4 gene in control and NS-MD populations.

AP2, 5-HTTLPR	Controls	MR patients	MR vs Controls	
			
			*χ*^2^	p
Haplotypes (5HTT gene)				
AL	179 (54.57)	110 (56,12)	0.12	0.73
AS, GL, GS	149 (45.43)	86 (43,88)		
				
Phased genotypes (5HTT gene)				
AL/AL	45 (27.44)	28 (28.57)	1.28	0.73
AL/AS	73 (44.51)	47 (47.96)		
AL/GL	16 (9.76)	6 (6.12)		
AL/GS	0 (0.0)	1 (1.02)		
Others	30 (18.29)	16 (16.33)		
				
Genotypes (5HTT/BDNF genes)				
AL AL/Val Val	19 (13.00)	10 (12.66)	0.65	0.88
AL AL/Val Met	21 (14.40)	11 (13.92)		
Others/Val Val	59 (40.40)	36 (45.57)		
Others/Val Met	47 (32.20)	22 (27.85)		

**Table 3:** Haplotypes and genotypes distributions for the VNTR and rs3813034 polymorphisms in the SLC6A4 gene in controls and NS-MD patients.

VNTR, rs3813034	Controls	MR patients	MR vs Controls	
			
			*χ*^2^	p
Haplotypes				
12G	125 (38.1)	70 (35.7)	3.8	0.28
12T	85 (25.9)	48 (24.5)		
10T	99 (30.2)	72 (36.7)		
Others	19 (5.8)	6 (3.1)		
				
Genotypes				
12G/10T	38 (23.0)	26 (26.2)	3.5	0.75
12G/12T	32 (19.8)	17 (17.6)		
12G/12G	24 (14.5)	13 (12.9)		
12T/10T	26 (15.7)	17 (17,8)		
12T/12T	11 (6.7)	6 (5.99)		
10T/10T	15 (9.1)	13 (13.3)		
Others	18 (11.2)	6 (6.1)		

### Analysis of the BDNF Val66 Met polymorphism in NS-MD

Hardy Weinberg distribution was respected for the alleles of the *BDNF *gene in the two populations. We developed a rapid genotyping method using dHPLC to analyze the allelic distribution of the rs6255 (Val66 Met) polymorphism in the *BDNF *gene (Figure 1). A first analysis by dHPLC allowed to identify the heterozygote genotypes (G/A), and a second analysis, in presence of a standard, allowed to discriminate between G/G or A/A homozygotes. We did not find a significant association between NS-MD and alleles of the rs6255 polymorphism (χ^2 ^= 0.40; p = 0.53) (Table 1).

Several studies have suggested a synergistic interaction between the serotonergic and the BDNF signalling systems [35]. We did not observe an association between NS-MD and the 5HTTLPR/rs6255, rs25531 (AP2)/rs6255, or rs3813034/rs6255 genotypes (data not shown). No association was found between NS-MD and a particular combination of AP2-5HTTLPR (AL or others) of the SLC6A4 gene and rs6255 (A or G) of the BDNF gene.

## Discussion

We here report the first genetic study on the serotonin transporter and the brain-derived neurotrophic factor genes in NS-MD. We have investigated five functional polymorphisms in these genes in control individuals and NS-MD patients matched for origin (Central France) and ethnicity (Caucasian).

Power calculations had shown that with the given sample sizes we should be able to detect differences in allelic frequencies of 12% for 5-HTTLPR, 12% for VNTR, 13% for rs3813034, and 10% for *BDNF *rs6255 (alpha of 5%, power of 80%). Functional studies have reported that the S allele of 5-HTTLPR was associated with a lower expression of 5-HTT and a lower serotonin reuptake activity [12,36,37]. Our results do not support a direct role for the S allele of 5-HTTLPR in NS-MD. Wendland et al. [32] suggested that the effect of 5-HTTLPR on *SLC6A4 *expression may be due to a nearby (19 bp) rs25531 polymorphism in the promoter. The G allele of this polymorphism is located in a consensus binding site for AP2, a family of transcription factors described as positive or negative regulators of transcription [38]. Data from Hu and colleagues [39] has shown that the G allele of rs25531 was associated with a decreased expression of 5-HTT mRNA compared to the A allele. We did not detect differences in rs25531 allelic frequencies between NS-MD and control patients. Moreover, we have observed that the frequency of the GS haplotype in both NS-MD and control patients was very low, suggesting that the G allele of rs25531 was not a risk factor for NS-MD. No statistically significant difference was observed between control and NS-MD patients for genotypes and allelic distributions of the VNTR and rs3813034.

Brain-derived neurotrophic factor (BDNF) is a key regulatory protein of serotonin levels in several brain regions. The pro-BDNF protein is cleaved to form a mature protein before being secreted. The rs6255 polymorphism (Val66 Met), which affects this cleavage, has been associated with impaired hippocampal functioning and decreased scores on the Logical Memory subtest of the Wechsler Memory Scale-Revised [28]. Moreover, Harris et al. [40] have shown that the rs6255 genotype contributes to age-related changes in reasoning skills, which are closely related to general intelligence. Thus, a number of studies support an important role for BDNF in cognition processes, and particularly in learning and memory. We did not observe an association between a particular allele or a genotype of rs6255 and French patients with NS-MD. However, it will be interesting to further analyze the complex BDNF locus in neurodevelopmental diseases such as NS-MD. Indeed, recent studies, which have reported a detailed characterization of the *BDNF *gene locus, have indicated 17 and 13 alternative transcripts for BDNF and anti-BDNF, respectively [41]. Anti-BDNF are antisense transcripts expressed from the opposite strand of the BDNF gene [26].

Serotonergic and BDNF signalling systems have significant interactions with overlapping functional targets [30]. Genetic epistasis between these two systems was confirmed by studies on *BDNF *and *SLC6A4 *transgenic mice [23,24]. In the present study on NS-MD patients we observed no genetic interactions between Val66 Met in *BDNF *and the four polymorphisms studied in *SLC6A4*. Particularly, we did not find a relation between the Met allele in BDNF and the S allele in SLC6A4, as described in children with high depressive scores [42]. However, these latter results obtained by Kauffman et al. [42] have been challenged by several studies reporting that the Met allele is protective for anxiety and depression [43,44].

## Conclusion

The present study suggests that the *SLC6A4 *and *BDNF *genes have no major effect on the aetiology of NS-MD. Moreover we did not find a gene-by-gene interaction in NS-MD patients between polymorphisms in the *BDNF *and *SLC6A4 *genes. However, further studies will be necessary to fully reject the implication of SLC6A4 and BDNF in NS-MD. Indeed the expression of these two genes is regulated by epigenetic processes, and these may still be impaired in NS-MD [45,46]. Moreover, effects on mRNA splicing, protein localization, and properties of 5-HTT and BDNF could be possible mechanisms for influencing NS-MD risk.

## Competing interests

The authors declare that they have no competing interests.

## Authors' contributions

RT carried out molecular genetic studies and contributed, with LM, to manuscript writing. SV, CCA, RAT and DDR participated in the molecular genetic studies. CRA, CM and PV participated in the design and the coordination of the study and manuscript writing. All authors read and approved the final manuscript.

## Pre-publication history

The pre-publication history for this paper can be accessed here:

http://www.biomedcentral.com/1471-2350/11/30/prepub
